# Hot Sulfur on the Rocks: The Reaction of Electronically Excited Sulfur Atoms with Water in an Ice-Surface Model

**DOI:** 10.1021/acsearthspacechem.4c00351

**Published:** 2025-03-25

**Authors:** Gabriella Di Genova, Jessica Perrero, Marzio Rosi, Cecilia Ceccarelli, Albert Rimola, Nadia Balucani

**Affiliations:** † Dipartimento di Chimica, Biologia e Biotecnologie, Università degli Studi di Perugia, 06123 Perugia, Italy; ‡ Departament de Quimica, Universitat Autònoma de Barcelona, 08193 Catalonia, Spain; § Dipartimento di Chimica and Nanostructured Interfaces and Surfaces (NIS) Centre, Università degli Studi di Torino, 10125 Torino, Italy; ∥ Dipartimento di Ingegneria Civile e Ambientale, Università degli Studi di Perugia, 06125 Perugia, Italy; ⊥ Univ. Grenoble Alpes, CNRS, Institut de Planétologie et d’Astrophysique de Grenoble (IPAG), 38100 Grenoble, France

**Keywords:** astrochemistry, DFT, interstellar medium, grain-surface chemistry, potential energy surfaces, kinetics

## Abstract

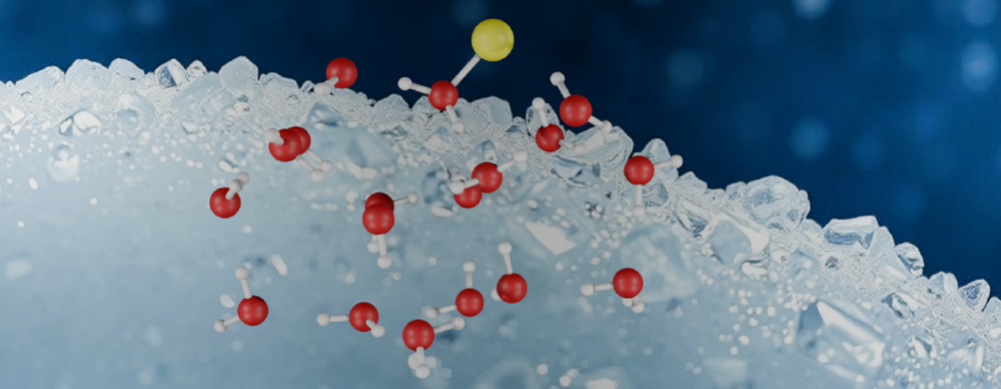

In this contribution, we present a theoretical investigation of the reaction involving atomic sulfur in its first electronically excited state, ^1^D, and H_2_O on an ice-surface model. This study is motivated by the work of Giustini et al. (*ACS Earth Space Chem.*, **2024**, *8*, 2318), which indicated a strong effect of the presence of four additional water molecules in the S(^1^D) + H_2_O reaction compared to the pure gas-phase case. Our simulation treats the long-range interactions (H-bonds and dispersion forces) with the ice water molecules in a much more realistic way being based on the use of a cluster of 18 water molecules, thus overcoming the limits of the small cluster used by Giustini et al. According to our results, S(^1^D) reacts via two possible reaction mechanisms: (1) addition to the O atom of a water molecule with the formation of H_2_OS or (2) insertion into one of the O–H bonds of a water molecule with the formation of HOSH. Both H_2_OS and HOSH are stabilized on ice by energy dissipation rather than isomerizing or dissociating into two products as seen in the gas-phase reaction. The interaction with surrounding water molecules affects the entire reaction pathway by stabilizing intermediate species, reducing some barriers, and impeding the only two-product open channel of the gas-phase reaction. S(^1^D) can be produced by UV-induced photodissociation of various precursor molecules on the surface of interstellar or cometary ice or by other high-energy processes induced by electrons or cosmic rays also in the ice bulk. Therefore, our results can be of help in elucidating the mysterious sulfur chemistry occurring in the icy mantles of interstellar grains or in cometary nuclei. Furthermore, this study demonstrates that the product branching ratios of gas-phase reactions should not be uncritically used in modeling interstellar ice chemistry.

## 1 Introduction

Sulfur is the tenth most abundant element in the Solar System and local interstellar medium (its abundance with respect to hydrogen^1^ is 1.4 · 10^–5^) and is ubiquitous in space, with S-bearing species detected in interstellar and intergalactic regions,^2,3^ on planet surfaces and atmospheres,^4−6^ on moons,^7,8^ and small bodies like comets, asteroids, and meteorites.^9−14^ It is also one of the six essential elements (HCNOPS) of life, being present (mainly in its reduced forms) in numerous biomolecules, including proteins, sugars, nucleic acids, vitamin cofactors, and metabolites.^15^ Therefore, it is of interest to understand the carriers of reduced sulfur to early Earth. In the interstellar medium (ISM), sulfur can be found in molecules - either in the gas phase (e.g., SO, H_2_S, CS, H_2_CS, and C_3_S^16^) or adsorbed in the icy mantles of interstellar dust grains (OCS and, possibly, SO_2_^17−19^) - and in the refractory material of grains (as, for instance, iron sulfide^20^) that eventually form planetesimals. According to the Cologne Database for Molecular Spectroscopy,^2^ 39 S-bearing molecules have been detected so far in ISM. Nevertheless, in molecular clouds and star-forming regions sulfur is significantly depleted from the gas phase (the “sulfur depletion problem”) with only 1% of the initial S-budget observed in its gaseous species.^16,21−25^ In these regions, S is mostly present in its ionized form (S^+^), which largely increases the sulfur binding energy to the grain surface as for all the other charged species.^26^ In addition, dust grains typically carry a negative charge^27^ so that their electrostatic attraction causes an even larger sticking coefficient on dust grains. As a consequence, it has been suggested that the missing sulfur is present in the ice covering the dust grains. Yet, the only two sulfur species detected so far on the icy mantles of interstellar grains account for ca. 5% of the S elemental budget^18,19^ and, contrary to what initially suggested, H_2_S is not the main S carrier on ice.^17,28^ The main carriers of sulfur in dense clouds remain unidentified. Refractory sulfur species, such as sulfur allotropes (S_*n*_, *n* = 3–8), iron sulfide, or ammonium sulfide have been invoked as the main sulfur reservoirs.^21,29−31^ Some clues on the sulfur carriers in star-forming regions are offered by pristine small bodies of our solar system, such as asteroids, meteorites, and comets. Formed in the outer protosolar disc, cometary nuclei are believed to be formed by pristine material^32,33^ and their composition is alleged to resemble that of interstellar dust grains (and their icy mantles).^9^ Cometary comae have been investigated by remote spectroscopy for some years and, after the first detection of atomic S and CS in the comet C/1975 West,^34^ other simple sulfur species (H_2_S, OCS, SO, SO_2_, S_2_, CS_2_, H_2_CS, and NS) have been identified.^9,35−42^ The Rosetta orbiter Spectrometer for Ion and Neutral Analysis (ROSINA) allowed the identification of other S-species in the quiet coma of the Jupiter family comet 67P/Churyumov-Gerasimenko.^43^ In addition to the usual S-bearing species, S_3_, S_4_, CH_3_SH, and C_2_H_5_SH/CH_3_SCH_3_^10,43,44^ were identified for the first time. Even more complex S-bearing species were detected during two episodes of enhanced dust emission (e.g., CH_3_OS, CH_4_OS, C_2_H_4_OS, C_3_H_8_OS)^45,46^ including those with gross formula H_2_SO and HSO.^46^ Finally, a rich inventory of S-compounds was compiled after the analysis of the surface material of Ryugu,^11,47,48^ similar to the one obtained by the analysis of meteorites like Murchinson and Allende.^12^ Clearly, the more complex S-species found in pristine small objects like comets and asteroids/meteorites are the result of some unidentified chemistry involving the simple S-molecules found in star-forming regions.

In this work, we consider the possible role of atomic sulfur in its first electronically excited state, ^1^D, in the chemical evolution of interstellar or cometary ice. The ^1^D state of S atoms has a large internal energy content (it lies 110.5 kJ mol^–1^ above the ground ^3^P state) and is a metastable state with a radiative lifetime of ca. 30 s.^49^ In extraterrestrial environments, it is produced in the gas phase by UV-induced photodissociation or electron impact-induced dissociation of several precursor molecules like OCS and CS_2_.^50^ Other precursors of S(^1^D) are H_2_S and SO_2_ via their photodissociation at λ ≤ 155 nm (including the Lyman– α wavelength).^51−53^ While gas-phase S(^1^D) has been spectroscopically identified in the coma of the comet 153P/Ikeya-Zhang,^54^ the production of S(^1^D) is also expected when photodissociation occurs for the same molecules residing on the ice surface as recently demonstrated for the analogous case of atomic oxygen in its ^1^D state by Bergner et al.^55,56^ who investigated the O(^1^D) reactions on methane ice, ethane ice, and acetylene ice. Methanol and formaldehyde were observed in the case of the reaction with solid methane, ethylene oxide, and acetaldehyde in the case of the reaction with ethane, and ketene in the case of the reaction with acetylene.^56^ Recently, models on interstellar ice chemistry^57,58^ and ice chemistry in cometary nuclei^59^ have started considering explicitly so-called photodissociation-induced (PDI) reactions, in which transient species are produced by UV photodissociation (or radiolysis induced by galactic cosmic rays (GCRs)) and immediately react with an adjacent molecule of the ice matrix. This approach, originally introduced to account for nondiffusive ice chemistry,^57^ has been extended to the case of O(^1^D) and also suggested for other species of the same kind, such as C(^1^D), by Carder et al.^58^

Several gas-phase reactions of S(^1^D) have been recently investigated and revealed its ability to insert into σ bonds (thus activating molecules that are not much reactive otherwise^49,60^) in addition to adding to the π-system of multiple bonds^61,62^ or lone pairs.^63^ This is significant because, unlike atomic sulfur in its ground electronic ^3^P state, which has low reactivity with closed-shell species, S(^1^D) reactive collisions can be crucial in forming organosulfur and other sulfur-bearing compounds in various environments.^60−62^ Since the main component of cometary and interstellar ice is water, according to the PDI scheme, once formed, S(^1^D) will react with the surrounding species, and water is by far the most probable candidate. Our simulation could also contribute to understanding sulfur chemistry in atmospheric water aerosols containing S(^1^D) precursors like OCS, the most abundant atmospheric sulfur species that photodissociates in the upper troposphere by absorbing photons in the window between the O_2_/O_3_ absorption bands and produce both S(^3^P) and S(^1^D).^64^

To assess the possible role of the S(^1^D) reaction with the water molecules of ice, we have simulated the reaction occurring on amorphous ice by considering a cluster of 18 water molecules holding several dangling oxygen and hydrogen atoms (the dangling OH ice feature was recently observed toward the dense cloud Chamaeleon I^65^) and characterized the potential energy surface (PES) of the S(^1^D) + H_2_O reaction on the cluster model to highlight the differences with the same reaction in the gas phase. The results are compared with those previously obtained by Giustini et al.,^63^ who investigated the gas-phase and S(^1^D) reactions with a cluster of five water molecules. In that study, Giustini et al.^63^ noted significant differences in the reaction mechanism caused by the presence of four additional water molecules with respect to the case of gas-phase reaction (see below). Those differences motivated the present study since an improved simulation of the amorphous water ice will allow for a more accurate assessment of the influence of the ice on the reaction outcome.

## 2 Methodology

### 2.1 Ice Model

**Figure 1:**
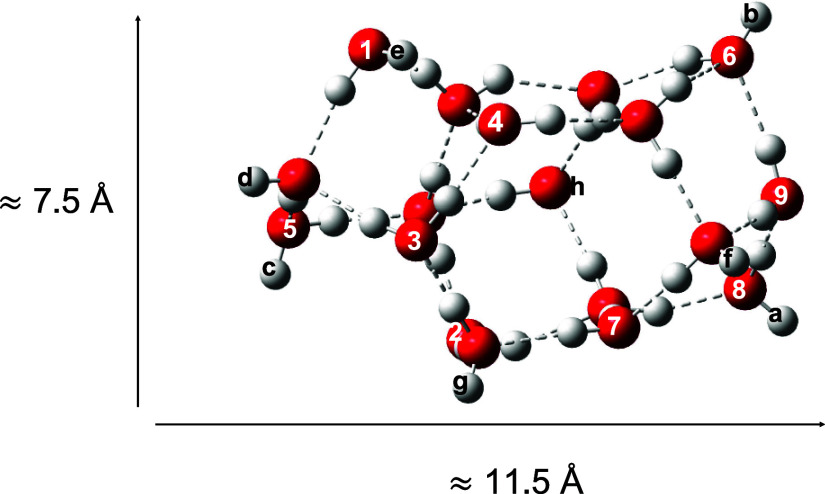
ωB97XD/ma-def2-TZVP-optimized structure for the water ice cluster model consisting of 18 water molecules (W_ice_).

Interstellar icy mantles were simulated with an 18-water cluster model (W_ice_, adopted in previous studies^66−69^) mimicking a compact, amorphous ice surface that presents flatter regions. The size of W_ice_ is a compromise between a realistic model (relatively large and amorphous) and the accuracy of the employed methodology, enabling the use of accurate, cost-effective, state-of-the-art theoretical methods without incurring high computational costs. The W_ice_ cluster model presents several adsorption sites, consisting of 8 dangling H atoms (dH, labeled as *a*–*f* in Figure 1) and 9 dangling O atoms (dO, labeled as 1–9 in Figure 1), showing a significant binding site variability, as expected for real amorphous water ice surfaces.

### 2.2 Electronic Structure Calculations

We employed gaussian16(70) and orca 6.0.1(71) software packages to carry out electronic structure calculations of the PESs for the S(^1^D)+H_2_O reaction in the gas phase and on W_ice_.

For the gas-phase reaction, given its reduced size, we performed geometry optimizations of the PES stationary points using the single- and double-electronic excitation coupled-cluster method with perturbative description of triple excitations, CCSD(T),^72−74^ in conjunction with the correlation consistent valence polarized basis set,^75−77^ augmented with a tight *d* function for the sulfur atoms to correct for the core polarization effects^78^ (aug-cc-pV(Q+d)Z). The energy of each stationary point was also computed at the CCSD(T)-F12 level of theory to compare them with previously published work. The F12 approximation allows for the computation of electronic correlation energies close to the limit of a complete basis set (CBS).^79^ The calculation requires a complementary auxiliary basis set (CABS) to factorize the many-electron integrals resulting from the use of explicitly correlated wave functions. This allows using medium-sized standard basis sets without compromising the quality of the results.^80,81^ Additionally, the PES was also computed at the domain-based local pair natural orbital coupled cluster theory with single-, double-, and perturbative triple-excitations DLPNO–CCSD(T) level of theory, in prevision of applying this methodology to W_ice_.^82^ When considering W_ice_, given the larger size of the system, geometry optimizations were performed at the DFT ωB97XD level of theory,^83^ in conjunction with the minimally augmented Karlsruhe basis set ma-def2-TZVP.^84^ The selection of the ωB97XD method was based on a preliminary benchmarking analysis, which allowed us to identify a suitable DFT functional that properly describes the electronic structure of the systems and reactions to study. Three DFT functionals were employed: BHLYP-D3(BJ), M062X-D3, and ωB97XD. The evaluation was based on comparing the DFT energy barriers against the values computed with single-point energy calculations at CCSD(T) level of theory on the optimized DFT geometries. This analysis was performed by considering the reactivity of S(^1^D) with a water molecule in the presence of a second water molecule assisting the reaction. The ωB97XD/ma-def2-TZVP methodology was used to compute the harmonic vibrational frequencies with the aim of checking the nature of the stationary points and deriving the vibrational zero point energy (ZPE) corrections. Minima, such as reactants, intermediates, and products, are characterized by positive eigenvalues of the Hessian matrix, while first-order saddle points, such as transition state structures, are identified by the presence of a single negative eigenvalue. Every energy accounts for the thermochemistry at 0 K, obtained by adding the ωB97XD-ZPE corrections onto the potential energies calculated at the different theory levels, this way deriving enthalpies at 0 K.

Intrinsic reaction coordinate (IRC)^85,86^ calculations were performed to link each identified saddle point with the respective reactant and product.

All the calculations involving one radical species were run as open-shell systems based on an unrestricted formalism. For those involving two radical species, we first optimized the structures as triplet states, followed by an open-shell singlet optimization to describe the biradical system. Singlet biradical systems were treated using the unrestricted broken symmetry approach,^87^ in which the most stable initial wave function was found using the *guess = mix* and *stable = opt* keywords in gaussian16.

At all the levels of calculation, the energy of the S(^1^D) electronic state was estimated by adding the experimental S(^3^P)-S(^1^D) separation, corresponding to 110.5 kJ mol^–1^,^49^ to the computed energy of the S(^3^P) state.

### 2.3 Kinetic Calculations

After calculating the energy barriers for the isomerization reactions (see Section 3.2) in the W_ice_ cluster model, we performed kinetic calculations to estimate the reactive lifetimes of the addition and insertion products. We used the Ramsperger-Rice-Kassel-Marcus (RRKM) theory^88^ to compute the reaction rate constant for the isomerization steps as a function of the total energy, *k*(*E*), using an in-house code^61,89,90^ adapted to the case of reactions on large clusters of water molecules.^67,68,91,92^ The *k*(*E*) for a certain reaction at a specific total energy is given by
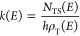
1where *N*_TS_(*E*) is the sum of states of the transition state at the total energy *E*, ρ_T_(*E*) is the reactant density of states at the total energy *E*, and *h* is the Planck’s constant. *N*_TS_(*E*) is obtained by integrating the relevant density of states up to energy *E*, assuming a rigid rotor/harmonic oscillator model. The density of states is symmetrized with respect to the number of identical configurations of the reactants and/or transition states. We treated each step as unimolecular and considered the whole cluster as isolated from its surroundings. Inside the isolated cluster, intramolecular vibrational redistribution is assumed to be fast. This approximation was verified to be good enough in other cases^67,91^ once we consider the time scale of energy randomization.^93,94^ From the unimolecular rate constants, we derived the lifetime of the reaction intermediates as the inverse of *k*(*E*) to evaluate if isomerization is competitive with the energy dissipation toward the entire ice matrix.

## 3 Results

### 3.1 Reaction S(^1^D) + H_2_O in the Gas Phase

**Figure 2:**
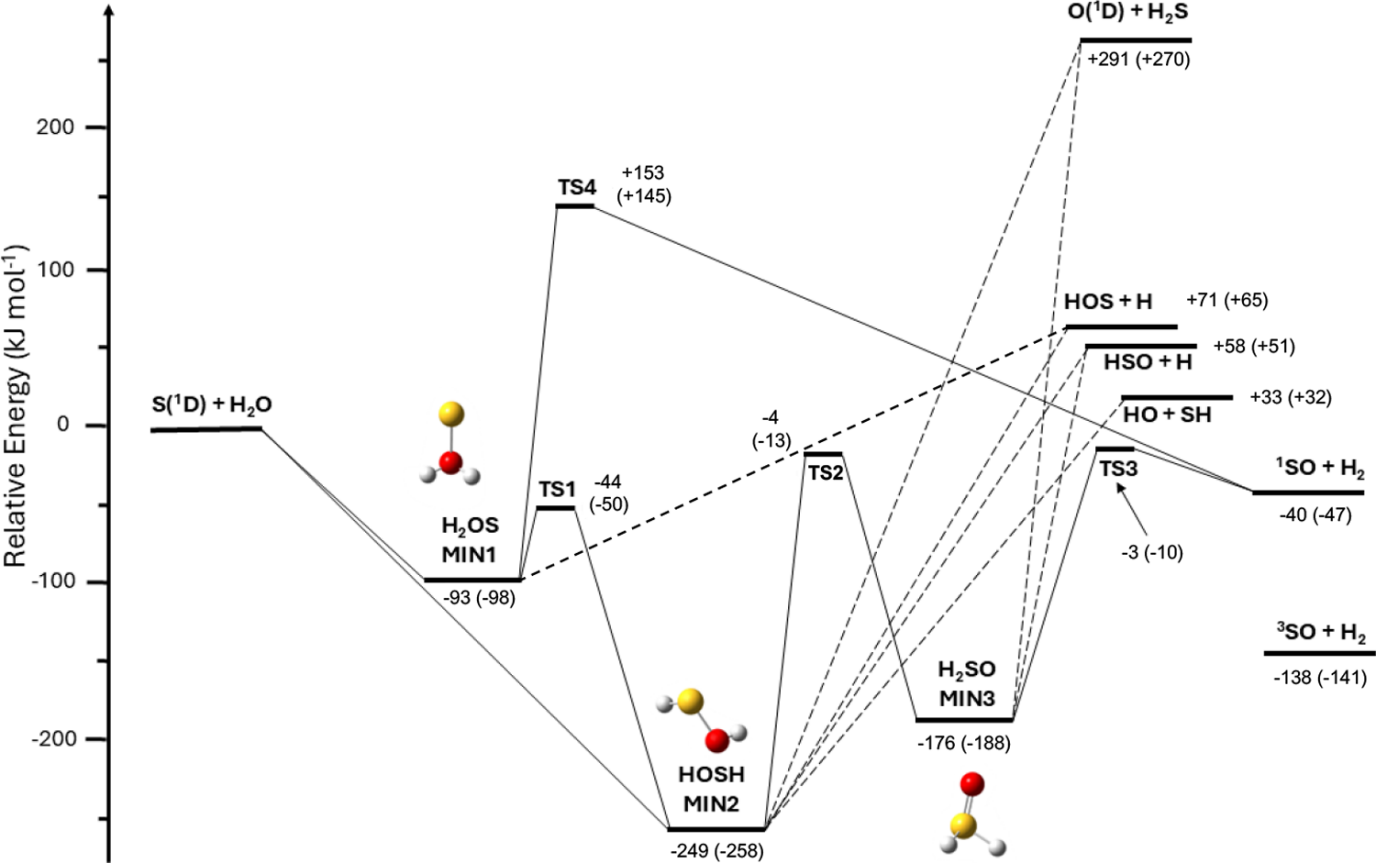
ZPE-corrected PES for the gas-phase S(^1^D) + H_2_O reaction channels. Geometries and energies are calculated at the CCSD(T)/aug-cc-pV(Q+d)Z level of theory. The values obtained at the CBS level of theory on optimized ωB97XD structures by Giustini et al.^63^ are also shown in parentheses for comparison.

The PES of the gas-phase S(^1^D) + H_2_O reaction, optimized at CCSD(T)/aug-cc-pV(Q+d)Z, is summarized in Figure 2. The energy values are reported rounded to the nearest kJ/mol considering the accuracy of the calculations. As already indicated by the calculations of Giustini et al.,^63^ S(^1^D) can either interact with one of the lone pairs of the oxygen atom, forming the addition intermediate H_2_OS (MIN1), or insert into one of the O–H bonds of the water molecule, forming the insertion intermediate HOSH (MIN2). Both entrance channels are barrierless. The H_2_OS intermediate is formed by electron charge transfer, mainly from the O atom of H_2_O to S, as indicated by the natural population analysis calculation (see Supporting Information, SI). This charge transfer is possible because S(^1^D) has two pure electronic microstates that present an empty 3p orbital to accommodate electron density. Once formed, H_2_OS can either isomerize to HOSH through TS1, by overcoming an intrinsic energy barrier of 49 kJ mol^–1^ (TS1 of Figure 2), or decompose into SO + H_2_ (since this is a singlet PES, the SO molecule is formed in its excited state a^1^Δ) by overcoming a very high intrinsic barrier of 246 kJ mol^–1^ (associated with a three-center elimination transition state, TS4 of Figure 2), or decompose into HOS + H in an endothermic channel. HOSH (MIN2) can either isomerize to MIN1 through the TS1 barrier, now of 205 kJ mol ^–1^, or isomerize to H_2_SO (MIN3) by overcoming an intrinsic barrier of 245 kJ mol ^–1^ (TS2), or fragment into OH + SH, HSO + H and HOS + H (all these dissociation channels are endothermic). MIN3 can isomerize back to MIN2 (now with a barrier of 174 kJ mol ^–1^, TS2), or decompose into HSO + H (an endothermic channel) or into SO(a^1^Δ) + H_2_ by overcoming a barrier (TS3) located at −3 kJ mol^–1^ with respect to the reactants asymptote. As already noted by Giustini et al. 2024, this is the only two-product open channel since it is both exothermic and characterized by submerged barriers along the energy path.

Table 1 compares the relative energies of the stationary points for the S(^1^D) + H_2_O PES with the values obtained by Giustini et al.^63^ at the CBS and CCSD(T)/aug-cc-pV(T+d)Z levels of theory on optimized ωB97XD structures. The results of the present work are in good agreement with the CBS data, the two methods lying within the chemical accuracy (4 kJ mol^–1^), with the only significant discrepancy associated with the energy of the O(^1^D) + H_2_S product channel. The agreement is less good with respect to the CCSD(T)/aug-cc-pV(T+d)Z calculations.^63^ Our data also nicely compare with the Δ*H*_0,*r*_^°^ = +30.7 kJ/mol for the OH + SH channel (derived by using the accepted values of Δ*H*_0,*f*_^°^ for OH and SH, see Appendix A5 of Atkinson et al.^95^). The agreement is also quite good when considering the difference in energy with respect to the SO product in its ground triplet state () which is - 143.75 kJ/mol (when using the values by Atkinson et al.^95^). However, the internal energy content of the excited SO(a^1^Δ) corresponds to 98 kJ/mol to be compared with the accepted value of 77.11 kJ/mol (*T*_0_ = 5861 cm^–1^, see Borin and Ornellas^96^). Therefore, our method, as well as those used by Giustini et al.,^63^ confirm their problems in dealing with electronic excitation.

**Table 1:** Relative Energies (in kJ mol^–1^) with Respect to the S(^1^D) + H_2_O Asymptote of the Stationary Points along the PES Shown in Figure 2^a^

	CCSD(T)/aug-cc-pV(Q+d)Z	DLPNO-CCSD(T)	CCSD(T)-F12	CBS	CCSD(T)/aug-cc-pV(T+d)Z
	this work	this work	this work	Giustini et al.^63^	Giustini et al.^63^
H_2_OS	–93	–93	–94	–98	–86
HOSH	–249	–248	–256	–258	–244
H_2_SO	–176	–176	–188	–188	–171
TS1	–44	–42	–46	–50	–39
TS2	–4	–5	–9	–13	+2
TS3	–3	–3	–8	–10	+5
TS4	+153	+154	+148	+145	+153
^3^OS + H_2_	–138	–139	–142	–141	–152
^1^OS + H_2_	–40	–40	–45	–47	–35
OH + SH	+33	+33	+31	+32	+31
HSO + H	+58	+57	+49	+51	+60
HOS + H	+71	+72	+64	+65	+73
H_2_S + O(^1^D)	+291	+291	+278	+270	+265

a The data at the CCSD(T)/aug-cc-pV(Q+d)Z level obtained in this work are shown in the first column, while the single energy point computed at the DLPNO–CCSD(T) and CCSD(T)-F12 level of theory are presented, respectively, in the second and third column. The fourth and fifth columns list the data obtained by Giustini et al.^63^ at the CBS and CCSD(T)/aug-cc-pV(T+d)Z levels on optimized ωB97XD structures for comparison.

### 3.2 Reaction of S(^1^D) with a Cluster of 18 Water Molecules

**Figure 3:**
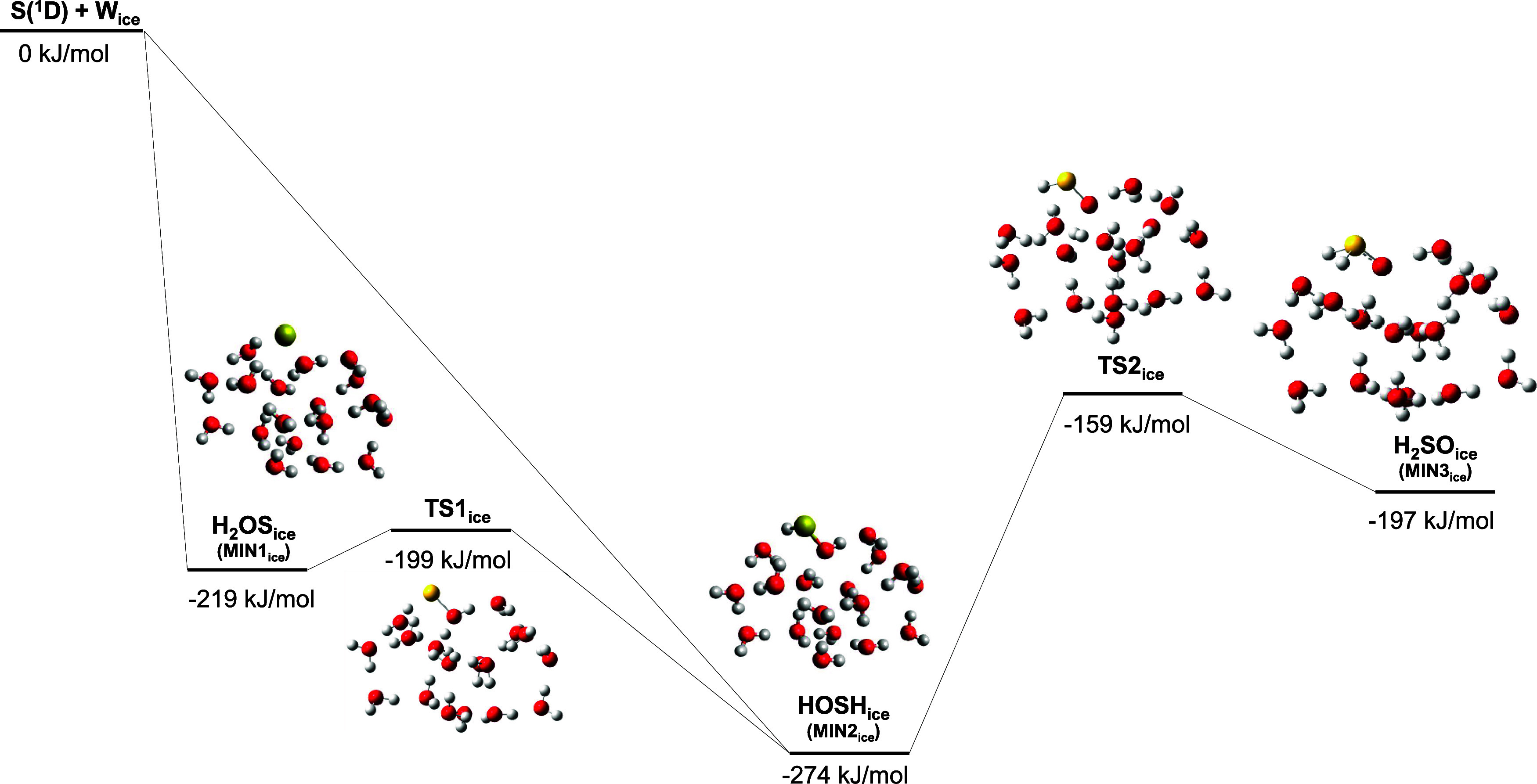
DLPNO–CCSD(T)/aug-cc-pVTZ//ωB97XD/ma-def2-TZVP PES, including ZPE corrections (at ωB97XD/ma-def2-TZVP), of the surface reaction of S(^1^D) + H_2_O. Energies are in kJ mol^–1^.

**Figure 4:**
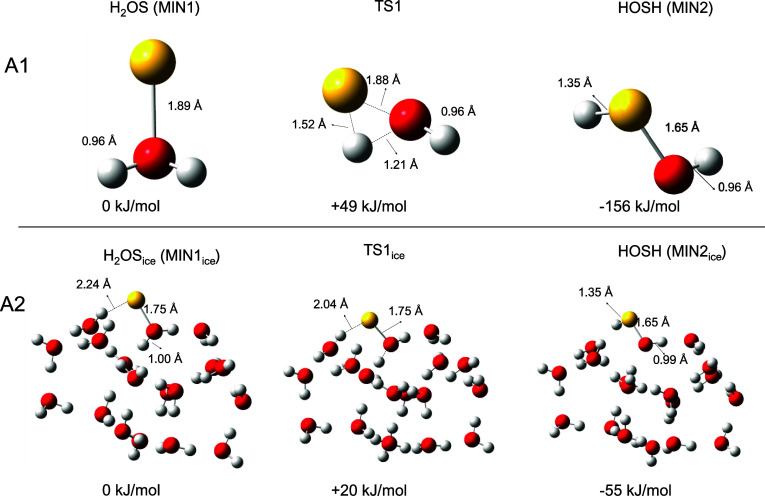
Optimized structures and relative energies (at the corresponding energy levels) of the stationary points for the S(^1^D) + H_2_O addition reaction in the gas phase (A1) and on W_ice_ (A2).

**Figure 5:**
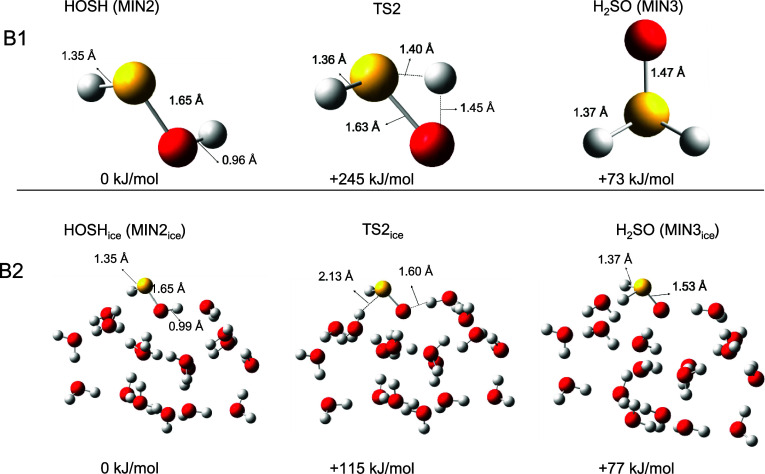
Optimized structures and relative energies (at the corresponding energy levels) of the stationary points for the S(^1^D) + H_2_O insertion reaction in the gas phase (B1) and on W_ice_ (B2).

According to our benchmarking analysis, the method yielding the smallest error on the energy barriers was ωB97XD (15%), followed by M062X-D3 (21%), and BHLYP-D3(BJ) (23%) (see SI). Therefore, ωB97XD was selected to perform our calculations on W_ice_. S(^1^D) was adsorbed on the 9 different dO positions of the W_ice_ cluster (see Figure 1), the dO4 binding site being the one providing the most favorable interaction energy (−219 kJ mol^–1^). The geometries and interaction energies of each binding site (dO1–9) are shown in SI. Figure 3 shows the PES of the S(^1^D) + H_2_O reaction on W_ice_, the geometries of the stationary points optimized at the ωB97XD level of theory and the energetics refined at DLPNO–CCSD(T). Bond distances for each stationary point are shown in Figures 4 and 5. As in the gas phase, the S(^1^D) atom can either add to one of the lone pairs of dO4 forming H_2_OS_ice_ (MIN1_ice_) or directly insert into one of the O–H bonds protruding from the cluster forming HOSH_ice_, (MIN2_ice_). The formation of H_2_OS_ice_ and HOSH_ice_ is highly exothermic (−219 kJ mol^–1^ and −274 kJ mol^–1^, respectively). HOSH_ice_ can also be formed via the isomerization of H_2_OS_ice_ via a barrier of 20 kJ mol^–1^. Finally, H_2_SO_ice_ can be formed by the isomerization of HOSH_ice_ but a barrier of 115 kJ mol^–1^ needs to be overcome.

The gas-phase and the ice-surface reaction pathways starting with the S(^1^D) addition to H_2_O forming H_2_SO and continuing with its isomerization into HOSH are compared in Figure 4, while the gas-phase and the ice-surface reaction pathways starting with the S(^1^D) insertion forming HOSH and continuing with its isomerization into H_2_SO are compared in Figure 5. The presence of the ice water molecules significantly stabilizes the addition product, with H_2_OS_ice_ being located at −219 and H_2_OS at −93 kJ mol^–1^ with respect to their respective reactants asymptotes. Such a large stabilization is due to the H-bonds that H_2_OS establishes with the water molecules of the ice surfaces. H-bond cooperativity in H_2_OS_ice_ is such that the S–O bond length decreases from 1.89 Å in H_2_OS to 1.75 Å in H_2_OS_ice_. HOSH_ice_ and H_2_SO_ice_ are also somewhat stabilized with respect to their gas phase counterparts by establishing H bonds with ice water molecules, but to a smaller extent (−274 vs −249 kJ mol^–1^ for HOSH_ice_ and HOSH, respectively; −197 vs −176 kJ mol^–1^ for H_2_SO_ice_ and H_2_SO, respectively). Therefore, H_2_OS_ice_ is the primary beneficiary of the presence of water ice, being highly stabilized compared to its gas-phase analog. The presence of the water ice surface also has important effects on energy barriers. Indeed, significant reduction of the energy barriers associated with the TS1_ice_ and TS2_ice_ (20 and 115 kJ mol^–1^) with respect to the TS1 and TS2 (48 and 246 kJ mol^–1^) is observed, which can be explained by the fact that the presence of the ice water molecules assists the H-transfer involved in the molecular rearrangement. Indeed, in TS1_ice_, three water molecules participate in the H-transfer mechanism, while in TS2_ice_, six water molecules are involved. In the gas phase, the TS1 and TS2 have a highly strained three-membered ring structure. In contrast, in W_ice_ the H-transfer mechanism assisted by water molecules relies on a TS structure less strained with larger rings corresponding to a lower barrier. The imaginary frequencies of each transition state are also reduced when moving from the gas-phase to W_ice_. The imaginary frequencies for TS1 and TS1_ice_ are 1565.32*i* cm^–1^ and 1001.10*i* cm^–1^, respectively, and for TS2 and TS2_ice_ are 1786.60*i* cm^–1^ and 701.89*i* cm^–1^.The displacement vectors for TS1_ice_ and TS2_ice_ are shown in Figure 1 of the SI. These decreases confirm the participation of more atoms in the TS_ice_ structures, with the lowering caused by the larger total mass of the atoms involved. Since the curvature along a specific direction on the PES is proportional to the eigenvalue of the Hessian matrix in that direction, which corresponds to the vibrational frequency of a mode, such decreases indicate that the degree of curvature of the potential barrier highest points becomes smoother on W_ice_, in line with the less-strained TS geometries.

Another significant effect is that we could not identify a TS connecting MIN3_ice_ to the SO(a^1^Δ) + H_2_ products. This can be easily justified considering that, for the gas-phase reaction, the H_2_ elimination occurs via a 3-center elimination mechanism that requires a large distortion of the structure of MIN3. The presence of H-bonds with the additional water molecules contrasts the necessary distortion of MIN3_ice_ and the only two-product exothermic channel leading to SO(a^1^Δ) + H_2_ channel is not open in this case. Also, the channel associated with the S–O bond cleavage from MIN2 does not seem to be open starting from MIN2_ice_ because of the additional resistance of the network of H-bonds to which HOSH belongs.

### 3.3 Kinetics of the Reaction

The rates of the unimolecular isomerization processes occurring on W_ice_ shown in Figure 3 were computed via the RRKM theory to estimate the average reactive lifetime of each species formed on the water ice. As commented above, these estimates are made by considering the cluster as an isolated system that cannot exchange energy or matter with the environment. Once the atom is adsorbed on the cluster, the reaction energy generated is retained as internal energy by the molecule. If the lifetime of this newly formed species is longer than 1 ps, the time in which almost all the reaction energy is dissipated throughout the ice surfaces,^93,94,97^ we can conclude that the isomerization process cannot take place. In contrast, if the lifetime is shorter, the internal energy can be used to overcome the isomerization energy barrier.

**Figure 6:**
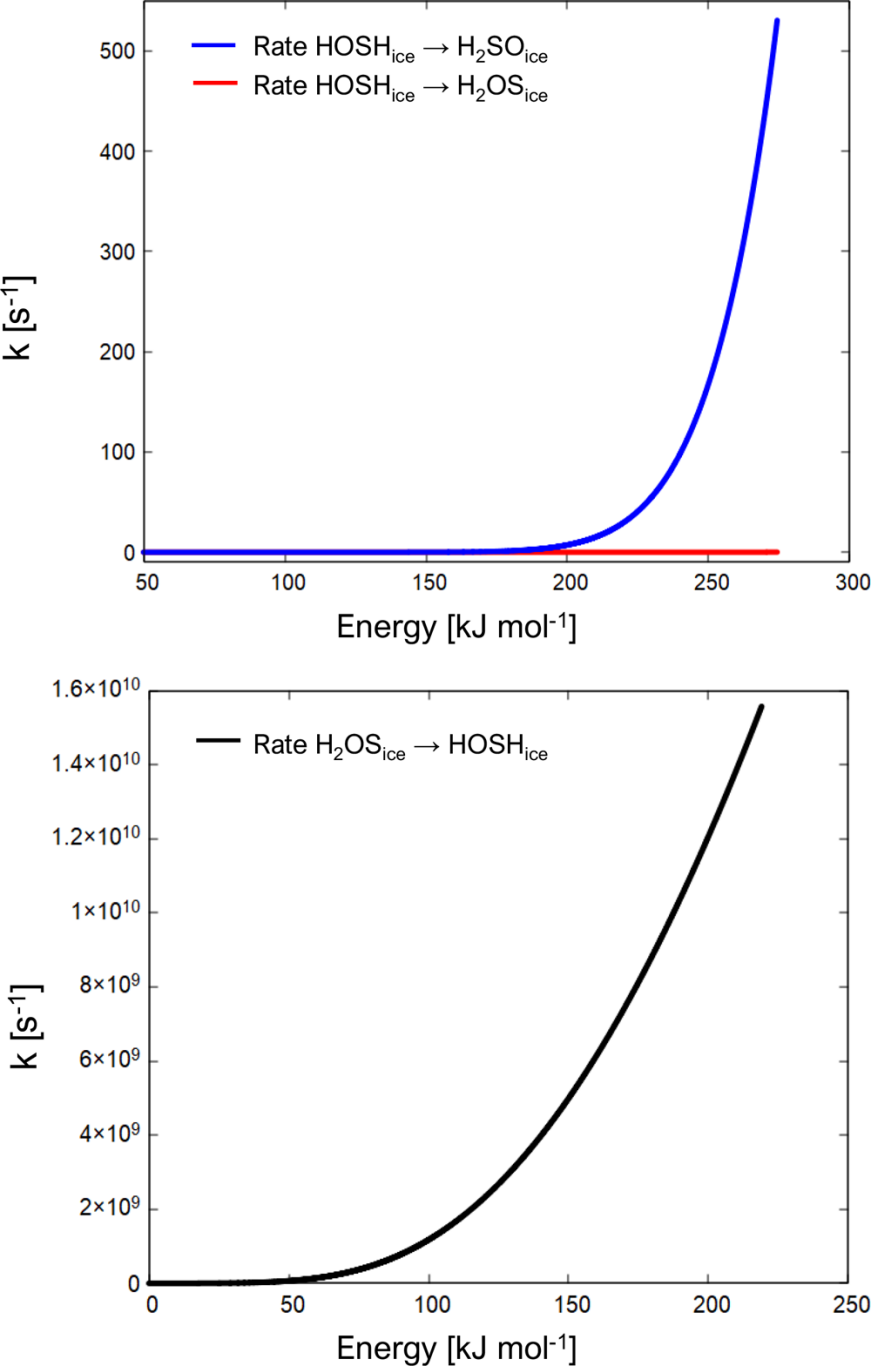
Unimolecular rate constants (s^–1^) for the conversion of HOSH_ice_ to H_2_OS_ice_ and HOSH_ice_ to H_2_SO_ice_ (upper panel) and H_2_OS_ice_ to HOSH_ice_ (bottom panel) as a function of the energy (kJ mol^–1^).

Figure 6 shows the unimolecular rate coefficient for the conversion of H_2_OS_ice_ to HOSH_ice_, as a function of the energy available to the system. The lifetime of the H_2_OS_ice_ species is the inverse of the unimolecular rate constant. The latter corresponds to the initial energy content deriving from the interaction of S(^1^D) with the water molecule of the ice surface (219 kJ mol^–1^). The calculated lifetime of H_2_OS_ice_ with respect to its isomerization is 64.2 ps. In the case of HOSH_ice_, the lifetime was calculated as the inverse of the sum of the unimolecular rate constants of the processes HOSH_ice_ → H_2_SO_ice_ and HOSH_ice_ → H_2_OS_ice_. Considering an initial energy content of 274 kJ mol^–1^, the lifetime of HOSH_ice_ is 0.19 ms.

Therefore, the estimated lifetimes of the H_2_OS_ice_ and especially of HOSH_ice_ (with respect to their isomerization processes) are much longer than the expected time scale in which the energy released by their formation is dissipated throughout the ice. According to ab initio molecular dynamics simulations (aimed at studying the fate of the energy released in exothermic reactions occurring in the presence of water ice), in 1 ps: (1) two-thirds of the energy liberated in the H + H recombination reaction (ca. 300 kJ mol^–1^) is absorbed by the ice,^94^ (2) 90% of the energy liberated in the H + CO → HCO reaction (ca. 130 kJ mol^–1^) is absorbed by the ice,^94^ and (3) about 60–90% of the energy released in the H additions to N up to the formation of NH_3_ is absorbed by the ice.^97^ Considering the large vibrational coupling with the water molecules of the present system, the energy dissipation is expected to occur within the same time scale, if not faster. Therefore, we speculate that isomerization does not occur appreciably, especially in the case of HOSH.

## 4 Discussion and Astrophysical Implications

The main motivation for the present study was the fact that by comparing the reaction mechanism of S(^1^D) + H_2_O in the gas-phase and in the presence of four additional water molecules significant differences were noted.^63^ However, the use of a minimal cluster like that provided by 4 water molecules (hereafter referred to as 4WMC, see Giustini et al.^63^) is rather simplistic and important surface effects could have been neglected. Our work aims at gaining deeper insights into the title reaction occurring on ice using a more realistic model of the water ice surface. As a reminder, the 4WMC PES was derived at the CCSD(T)/ωB97XD level, while our results were obtained using the W_ice_ model at the CCSD(T)/ωB97X-D3 level. Therefore, the comparison is highly consistent as far as the methodology is concerned.

Based on our results, the effects arising from W_ice_ are even more significant than those using the minimal 4WMC. More specifically: (1) the relative energies (with respect to their asymptotes) of H_2_OS and H_2_OS_ice_ are −93 and −219 kJ mol^–1^, respectively, while in the 4WMC model they changed only from −86 to −161 kJ mol^–1^. This implies a significant extra-stabilization when long-range interactions (i.e., H-bond cooperativity and dispersion forces) are better taken into account. (2) the intrinsic energy barriers are 20 kJ mol^–1^ for TS1_ice_ vs 111 kJ mol^–1^ for TS1_4WMC_, and 115 kJ mol^–1^ for TS2_ice_ vs 244 kJ mol^–1^ for TS2_4WMC_. Clearly, the 4WMC model, due to its small size, cannot take into account all the stabilizing H-bonds in the TS structures. Moreover, the number of water molecules involved in the water-assisted H-transfers is limited to one on the 4WMC model for both TSs, while on W_ice_ more water molecules are involved. As already commented on, this has the important consequence of strongly reducing the geometrical strain of the TSs, and hence an enhanced decrease of the associated energy barriers. In fact, with 4WMC, only one water molecule assists the proton transfer process, forming a six-member ring, whereas in the presence of W_ice_, for TS1_ice_, three water molecules assist the process, forming a 10-member ring. Minor differences are instead noted for HOSH and H_2_SO, the relative energies of which are −249 for HOSH vs −274 kJ mol^–1^ for HOSH_ice_ (to be compared with the Giustini et al. values of −244 for HOSH and −250 kJ mol^–1^ for HOSH_4WMC_), and −176 for H_2_SO vs −197 kJ mol^–1^ for H_2_SO_ice_ (to be compared with the Giustini et al. values of −171 for H_2_SO and −180 kJ mol^–1^ for H_2_SO_4WMC_). This means that the stabilization of these two species is similar irrespectively of the water ice cluster model, as both water clusters can hold similar H-bond interactions with them. Finally, the only open two-product channel for the gas-phase reaction is not active on ice since H_2_SO_ice_ is never formed and the critical geometry of the 3-center elimination transition state could not be reached because the H-bonds that H_2_SO_ice_ forms with the surrounding water molecules will impede it.

Despite these differences, our work corroborates the suggestion put forward by Giustini et al. We verified that, once formed by the reaction of S(^1^D) with the water ice molecules, H_2_OS_ice_ and HOSH_ice_ will be stabilized by the ice. By comparing the reaction lifetimes in the most favorable scenarios (all the energy retained among the cluster molecules) with the energy dissipation time scale consistently derived in three different cases by using ab initio molecular dynamics, we can conclude that isomerization will not be significant.

H_2_OS_ice_ and HOSH_ice_ can accumulate on the ice surface or in the ice bulk, especially in the case of HOSH (hydrogen thioperoxide), the most stable H_2_SO isomer. H_2_OS_ice_ can react on the icy surface of interstellar grains with H atoms ultimately being transformed into HOSH (for a similar process, see Góbi et al.^98^). HOSH could undergo the typical interstellar ice reactions (namely, hydrogenation), but for this species, we expect a significant barrier, as already observed for the similar species H_2_O_2_, whose reaction with H is characterized by a very large barrier (ca. 27 kJ mol^-^^1^), which increases when adding 3 water molecules.^99^ Even considering the tunneling effect at the very low temperature of interstellar ice, a significant portion of HOSH is expected to remain unchanged, as demonstrated by ice models in the case of H_2_O_2_.

Our work also supports the modeling approach by Garrod^59^ and Carder et al.,^58^ who were the first to explicitly consider PDI reactions on ice. Once formed by the photodissociation (or GCR radiolysis) of their precursors, very reactive species like S(^1^D) or O(^1^D) will immediately react with the surrounding molecules. Water is the best candidate, being the most abundant species in interstellar or cometary ice by far. The radiative lifetime of O(^1^D) was estimated to be 1 s in solid matrices. Considering that the lifetime of O(^1^D) is ca. 3 times larger than that of S(^1^D) in the gas phase, we expect that the lifetime of S(^1^D) in ice will be a fraction of one second. During this time, it reacts with water and forms either H_2_OS or HOSH. Notably, HOSH and H_2_SO can also form by the reaction of O(^1^D) if produced by UV photodissociation of H_2_O/CO_2_/CO in the vicinity of one H_2_S molecule.^100,101^ To the best of our knowledge, HOSH has never been detected so far in the interstellar medium, even though its presence has been already speculated.^102,103^ A related species, the HSO radical, was recently detected toward several cold dark clouds by using the Yebes 40 m telescope.^104^ HSO resulted to be a widespread molecule in cold regions, but its formation mechanism is still obscure.^104^ The gas-phase formation routes suggested so far are either endothermic (like the reaction between OH and SH, see Figure 2) or characterized by a significant barrier (like the O + H_2_S reaction^101^) while no formation routes from ion–molecule reactions have been envisaged.^104^

Our results can also contribute to explaining the presence of a peak associated with H_2_SO (and HSO) in the mass spectrum recorded by ROSINA on September 5, 2016 after a chunk of ice and dust hit the instrument. In that occasion, sublimation of less volatile species occurred inside the equilibrium chamber of ROSINA at a temperature of 273 K, so increasing the signal of all the sulfur species already detected and causing the appearance of new species in the mass spectrum. It is unclear if molecules with this formula were present in the ice or if the signal at these masses originated by dissociative ionization of larger molecules in the mass spectrometer. However, the signal recorded at *m*/*z* = 50 attributed by Mahjoub et al.^46^ to H_2_SO^+^ can plausibly be associated with the electron impact ionization of HSOH. The ice reaction investigated in this manuscript can be responsible for the formation and accumulation over time of HOSH that, being involved in strong H-bonds with the water molecules of ice, is not easily released in the gas phase. Notably, the similar species H_2_O_2_, rarely identified in the ISM, has been detected in the coma of the 67P comet.^105^

To conclude, a more general remark is in order. In the ice models by Carder et al.,^58^ the branching ratios of the gas-phase reactions have been adopted also for the O(^1^D) reactions on ice (except for the reaction with CH_4_, for which the value of Bergner et al.^55^ was used). This is a common approach when the data necessary to model ice chemistry are missing. However, our results clearly indicate that this approximation can cause serious mistakes. The presence of water molecules can impede the reaching of critical configurations for the reaction to occur while the formation of two products can be seriously disfavored by the presence of H-bonds. In most cases, if a closed-shell polar molecule is formed by the reaction of the electronically excited singlet species, it will easily be stabilized by energy dissipation.

## 5 Conclusions

In this work, we have investigated the S(^1^D) + H_2_O reaction occurring on an ice surface model of 18 water molecules using electronic structure and kinetics calculations. As in the gas-phase reaction, the initial approach of S(^1^D) can occur via the addition to the lone pair of an O atom of water (H_2_OS is formed), or via insertion into the O–H σ bonds of water (HOSH is formed). The initial approaches are the same as seen for the gas-phase reaction. However, on the water ice cluster, the reaction stops at the formation of H_2_OS and HSOH. For this specific reaction, we can clearly observe all the effects that water ice can exert: as a concentrator/supplier of reactants, as a third body that stabilizes the products formed, and as a chemical catalyst that provides alternative routes with lower energy barriers. Indeed, the ice surface stabilizes the newly formed products by acting as an energy dissipator thus preventing them from dissociating into two products or isomerizing. The catalytic effect exerted by the ice surface is also very clear: the energy barriers along the minimum energy path are significantly lowered because of the stabilization of the TS structures by H-bonding within the structures, and the occurrence of water-assisted H-transfer mechanisms.

Our results can be of help in elucidating the mysterious sulfur chemistry occurring in the icy mantles of interstellar grains or in cometary nuclei. We suggest that HOSH accumulates in interstellar ice. Furthermore, this study demonstrates that the product branching ratios of gas-phase reactions should not be uncritically used in modeling interstellar ice chemistry.

## Supporting Information Available

Natural population analysis; attack sites; displacement vectors for TS1_ice_ and TS2_ice_; energetics and errors of the benchmarking study; and optimized Cartesian coordinates (PDF)
