# Supplementary material for: Hot Sulfur on the Rocks: The Reaction of Electronically Excited Sulfur Atoms with Water in an Ice-Surface Model
Source: ACS Earth Space Chem. 2025 Mar 25;9(4):844–55. doi: 10.1021/acsearthspacechem.4c00351 (PMC12010427; doi:10.1021/acsearthspacechem.4c00351)
Supplement: Supplementary file 1 — sp4c00351_si_001.pdf [file sp4c00351_si_001.pdf]

Hot sulfur on the rocks: the reaction of electronically excited sulfur atoms with water in an ice-surface model

Gabriella Di Genova [*,†], Jessica Perrero [‡,¶], Marzio Rosi [§], Cecilia Ceccarelli [∥], Albert Rimola [*,‡] and Nadia Balucani [*,†]

†*Dipartimento di Chimica, Biologia e Biotecnologie, Università degli Studi di Perugia, 06123 Perugia, Italy*

‡*Departament de Quimica, Universitat Autònoma de Barcelona, 08193 Catalonia, Spain*

¶*Dipartimento di Chimica and Nanostructured Interfaces and Surfaces (NIS) Centre, Università degli Studi di Torino, 10125 Torino, Italy*

§*Dipartimento di Ingegneria Civile e Ambientale, Università degli Studi di Perugia, 06125 Perugia, Italy*

∥*Univ. Grenoble Alpes, CNRS, Institut de Planétologie et d'Astrophysique de Grenoble (IPAG), 38100 Grenoble, France*

E-mail: gabriella.digenova@dottorandi.unipg.it; albert.rimola@uab.cat; nadia.balucani@unipg.it

Natural Population Analysis

Table S1: Natural Population Analysis at ωB97X-D3/ma-def2-TZVP of the isolated H_2OS molecule.

Atom	Natural Charge	Core	Valence	Rydberg	Total
**O**	-0.737	2.000	6.721	0.017	**8.737**
H	0.524	0.000	0.473	0.003	0.476
H	0.524	0.000	0.473	0.003	0.476
**S**	-0.310	10.000	6.289	0.021	**16.310**
Total	0.000	12.000	13.955	0.045	26.000

Table S2: Natural Population Analysis at ωB97XD/ma-def2-TZVP of the H_2OS molecule on the surface of the W_{ice} grain.

Atom	Natural Charge	Core	Valence	Rydberg	Total
O	-1.004	2.000	6.990	0.014	9.004
O	-1.022	2.000	7.005	0.018	9.022
O	-1.010	2.000	6.993	0.018	9.010
O	-0.978	2.000	6.961	0.017	8.978
O	-0.976	2.000	6.960	0.016	8.976
O	-1.013	2.000	6.998	0.015	9.013
O	-1.005	2.000	6.989	0.016	9.005
**O**	-0.802	2.000	6.787	0.015	**8.802**
O	-0.973	2.000	6.957	0.015	8.973
O	-0.998	2.000	6.982	0.016	8.998
O	-1.016	2.000	7.000	0.017	9.016
O	-0.997	2.000	6.981	0.016	8.997
O	-0.960	2.000	6.946	0.015	8.960
O	-1.000	2.000	6.984	0.016	9.000
O	-1.034	2.000	7.015	0.018	9.034
O	-1.018	2.000	7.003	0.015	9.018
O	-0.991	2.000	6.977	0.014	8.991
O	-0.986	2.000	6.971	0.016	8.986
H	0.475	0.000	0.522	0.003	0.525
H	0.510	0.000	0.486	0.004	0.490
H	0.497	0.000	0.499	0.003	0.502
H	0.498	0.000	0.498	0.003	0.502
H	0.535	0.000	0.461	0.004	0.465
H	0.530	0.000	0.466	0.004	0.470
H	0.497	0.000	0.500	0.003	0.503
H	0.500	0.000	0.497	0.003	0.500
H	0.518	0.000	0.478	0.003	0.481
H	0.502	0.000	0.495	0.003	0.498
H	0.506	0.000	0.491	0.003	0.494
H	0.505	0.000	0.492	0.003	0.495
H	0.512	0.000	0.484	0.004	0.487
H	0.484	0.000	0.513	0.003	0.516
H	0.511	0.000	0.485	0.003	0.489
H	0.483	0.000	0.514	0.003	0.516
H	0.512	0.000	0.484	0.003	0.487
H	0.508	0.000	0.488	0.003	0.492
H	0.517	0.000	0.480	0.003	0.483
H	0.508	0.000	0.488	0.004	0.492
H	0.512	0.000	0.484	0.004	0.488
H	0.487	0.000	0.511	0.003	0.513
H	0.502	0.000	0.494	0.003	0.497
H	0.488	0.000	0.505	0.007	0.512
H	0.474	0.000	0.523	0.003	0.526
H	0.506	0.000	0.490	0.004	0.494
H	0.513	0.000	0.483	0.004	0.487
H	0.481	0.000	0.516	0.003	0.519
H	0.516	0.000	0.479	0.004	0.484
H	0.508	0.000	0.489	0.003	0.492
H	0.490	0.000	0.504	0.006	0.510
H	0.499	0.000	0.498	0.003	0.501
H	0.521	0.000	0.475	0.004	0.479
H	0.483	0.000	0.514	0.002	0.517
H	0.509	0.000	0.488	0.003	0.491
H	0.509	0.000	0.486	0.004	0.490
**S**	-0.327	10.000	6.300	0.027	**16.327**
Total	0.000	45.996	149.563	0.440	196.000

Energetics and errors of the benchmarking study

Table S3: Benchmark performed on a system composed by 2 water molecules and a S(^{1}D) atom. Each structure was optimized at 3 different levels of theory: ωB97XD/ma-def2-TZVP, M062X-D3/ma-def2-TZVP, BHLYP-D3(BJ)/ma-def2-TZVP. After each geometry optimization a single point calculation at the CCSD(T)/aug-cc-pV(T+d)Z level of theory. Each energy is corrected with the ZPE.

**Str.**	**E (Ha)**	**Rel. E (kj mol-1)**	**S.P. CCSD (Ha)**	**Rel. E (kJ mol-1)**	**Abs. Err.**	**Barriers Err.**
**DFT method: ωB97XD**						
MIN1	-550.961	0.0	-550.311	0.0		
MIN2	-551.008	-121.5	-550.355	-116.3	5%	
MIN3	-550.987	55.2	-550.331	62.6	12%	**15%**
TS1	-550.957	13.0	-550.304	17.0	23%	
TS2	-550.960	124.5	-550.304	133.2	7%	
**DFT method: M062X-D3**						
MIN1	-550.930	0.0	-550.311	0.0		
MIN2	-550.972	-111.5	-550.354	-114.7	3%	
MIN3	-550.947	65.4	-550.330	61.9	6%	**21%**
TS1	-550.925	11.1	-550.304	18.0	38%	
TS2	-550.923	127.9	-550.304	132.3	3%	
**DFT method: BHLYP-D3(BJ)**						
MIN1	-550.930	0.0	-550.309	0.0		
MIN2	-550.976	-119.3	-550.353	-116.2	3%	
MIN3	-550.953	59.2	-550.329	62.2	5%	**23%**
TS1	-550.921	24.9	-550.302	17.9	39%	
TS2	-550.921	142.1	-550.302	133.0	7%	

Attack sites

Attack site	Initial guess	Final structure	Interaction energy
dO1	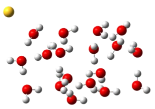	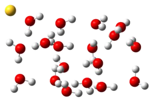	-131 kJ/mol
dO2	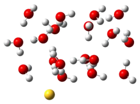	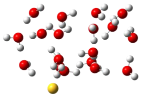	-173 kJ/mol
dO3	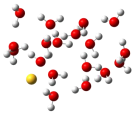	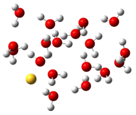	-168 kJ/mol
dO4	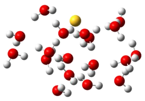	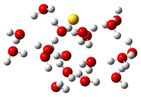	-219 kJ/mol
dO5	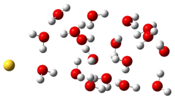	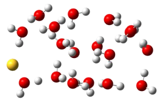	-151 kJ/mol
dO6	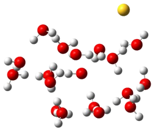	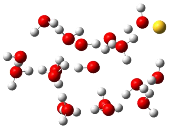	-158 kJ/mol
dO7	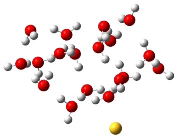	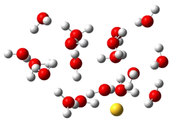	-181 kJ/mol
dO8	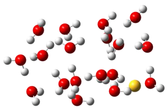	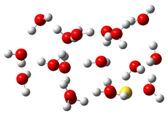	-165 kJ/mol
dO9	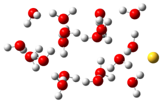	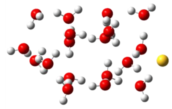	-164 kJ/mol

Figure S1: Geometries of the S atom adsorbed on each of the 9 dO9 sites of the W_{ice} cluster. Each structure has been optimized at the ωB97XD/ma-def2-TZVP level of theory. Interaction energies, computed at the DLPNO-CCSD(T) level of theory, are listed in the last column.

Displacement vectors for $TS1_{ice}$ and $TS2_{ice}$

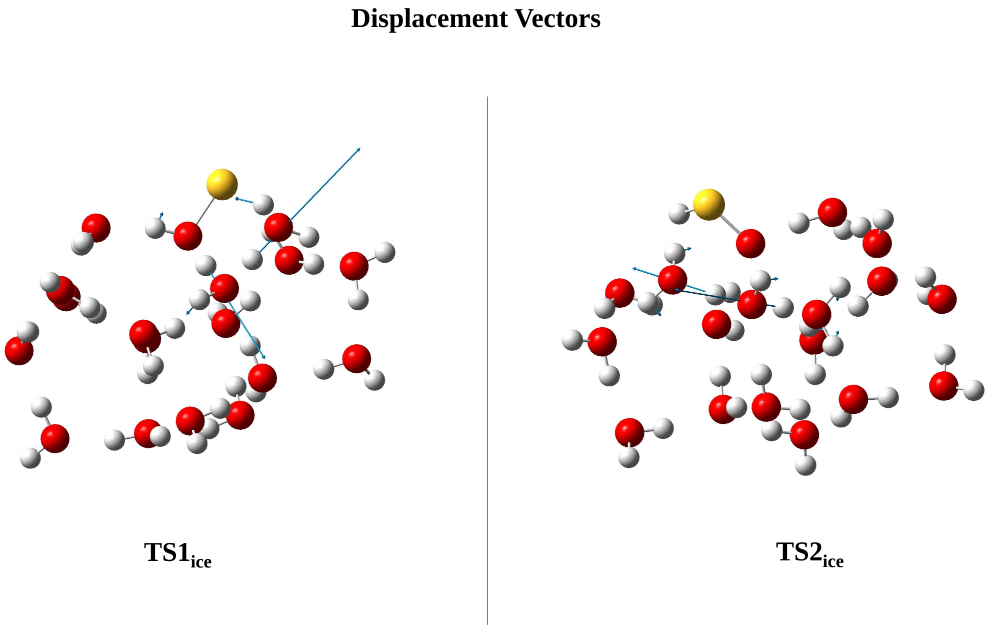

Figure S2: Displacement vectors for $TS1_{ice}$ and $TS2_{ice}$.

Optimized cartesian coordinates

H_2O

O 0.00000000 -0.11760000 0.00000000

H 0.75751600 0.47039900 0.00000000

H -0.75751600 0.47039900 0.00000000

H_2OS

O 1.15292900 -0.00005200 -0.11296500

H 1.42008200 -0.77509700 0.39679000

H 1.41823500 0.77612100 0.39621100

S -0.75385900 -0.00003800 0.00692000

HOSH

O -1.08264600 0.02047800 -0.11768400

H 0.86311300 1.22449600 0.00271500

H -1.43373400 0.03902800 0.77747100

S 0.57698700 -0.08920900 0.01008000

H_2SO

O -1.06163000 -0.00004600 0.06544900

H 0.95615600 0.95206800 0.70419700

H 0.95878200 -0.94848600 0.70724200

S 0.41113100 -0.00020100 -0.12093900

H_2

H 0.00000000 0.00000000 0.37099500

H 0.00000000 0.00000000 -0.37099500

OS

O 0.00000000 0.00000000 -0.99860200

S 0.00000000 0.00000000 0.49930100

OH

O 0.00000000 0.00000000 0.10784800

H 0.00000000 0.00000000 -0.86278700

SH

H 0.00000000 0.00000000 -1.26376300

S 0.00000000 0.00000000 0.07898500

HSO

O 0.05294200 1.03323400 0.00000000

H -1.27061300 -0.80967600 0.00000000

S 0.05294200 -0.46601200 0.00000000

HOS

O 0.03668300 1.03513000 0.00000000

H -0.88039300 1.33334800 0.00000000

S 0.03668300 -0.60089900 0.00000000

H_2S

H 0.96550500 -0.82388400 0.00000000

H -0.96550500 -0.82394700 0.00000000

S 0.00000000 0.10298900 0.00000000

TS1

O -1.19311400 0.02302300 -0.11547800

H -0.47322900 0.81736000 0.44850700

H -1.44868200 -0.61160100 0.56832600

S 0.71667600 -0.02437200 -0.00581300

TS2

O -1.13103100 0.04408500 -0.06588000

H 0.96484400 1.19078300 0.03733500

H -0.27596700 -0.07060300 1.11215700

S 0.52246100 -0.09205400 -0.03890300

TS3

O 1.06184800 0.19929500 -0.00007300

H -1.39820800 1.08224700 0.43006800

H -1.40140400 1.08325800 -0.42954600

S -0.35594800 -0.23499200 0.00000400

TS4

O 0.90841900 -0.25523000 -0.03345700

H 1.54569100 0.37243400 0.54859200

H 1.96789300 0.91003600 -0.30060500

S -0.67380900 0.04746100 0.00122900

W_{ice}

O 0.78604000 -1.12789300 -2.72516700

O -1.76876300 -1.36832000 -1.89602300

O -1.52296000 1.29506900 -1.40600600

O -3.60279700 2.83861100 -0.62219900

O -0.99289200 -3.01885200 0.08585300

O -1.31412400 -1.81311400 2.59256700

O -2.79897100 0.35874200 2.38809800

O -1.24888200 2.31551700 1.19232400

O -4.48640700 -1.55238300 -1.43537300

O -4.81999700 0.45563900 0.35680400

O 1.23853700 1.49415800 -1.82340000

O 3.61061500 2.71975800 -1.22524200

O 1.57084800 2.34827000 0.84328000

O 2.94679500 0.62849700 2.45340600

O 1.44459600 -1.47320400 2.14661200

O 1.76617300 -2.46256000 -0.42194200

O 4.43817300 -2.07333200 -0.76302900

O 4.67684900 0.45370900 0.21136100

H 5.19510300 -2.57324900 -0.45898800

H 4.56809200 -1.15208000 -0.44660500

H 4.17045200 0.52484800 1.03642100

H 4.42450300 1.22241000 -0.32188900

H -0.32176600 2.55235700 1.33896600

H -1.22967000 1.85163900 0.33721900

H -0.03562200 -3.13859900 0.00510700

H -1.14926100 -2.69285400 0.98899500

H 2.74160100 -2.50766400 -0.49201200

H 1.48665400 -1.98759000 -1.22132400

H 2.24180600 2.91949700 0.45154400

H 2.04517900 1.79859900 1.49864400

H 2.36897500 -0.18426500 2.41599500

H 3.23128600 0.74114300 3.36013700

H 2.86124100 2.33668900 -1.72093900

H 4.08630300 3.29793900 -1.82151900

H 1.21603000 0.57128400 -2.13280600

H 1.24011700 1.47874000 -0.84974400

H 0.50058700 -1.51047300 2.37208600

H 1.52801900 -1.79484800 1.22826200

H -0.18072600 -1.28907000 -2.62474800

H 1.05589700 -1.48547300 -3.57158900

H -3.52719500 0.34681700 1.74708000

H -2.22654000 1.10330000 2.12260200

H -4.66184700 -2.45521300 -1.16730700

H -3.55749100 -1.54651000 -1.74565400

H -4.73259200 -0.28297300 -0.28974800

H -5.74592900 0.49462800 0.59978600

H -2.26230000 1.93632400 -1.42895700

H -0.74097500 1.65933600 -1.84611200

H -3.11762500 3.16099000 0.14556500

H -4.13526700 2.09508100 -0.29276600

H -1.89669400 -1.01013400 2.52550600

H -1.57534800 -2.27998200 3.38664500

H -1.73526600 -0.42765800 -1.62552500

H -1.51240400 -1.91947100 -1.11772500

H_2OS_{ice}

O -1.04494700 2.96477200 -1.80307000

O 1.34269600 2.50657700 -0.64758200

O 1.20195200 -0.21349100 -1.14551100

O 3.39415800 -1.37880200 -1.93082000

O 0.43334500 3.04828900 1.83073900

O 0.94536000 0.40047700 2.55827700

O 3.19232400 -0.67996200 1.74536000

O 0.65361900 -2.23271400 0.40956900

O 4.07130000 2.91435100 -0.78434000

O 4.90722800 0.38296700 -0.27525700

O -1.43191200 0.19516600 -1.76183900

O -3.12965800 -1.83040600 -2.19586100

O -1.62427100 -3.19307000 -0.21235600

O -3.05965100 -1.79046000 1.72331900

O -1.35139800 0.16266800 1.03976000

O -2.23154700 2.70781000 0.75769000

O -4.77605900 1.95399400 0.26082600

O -4.86703900 -0.72609500 -0.17289400

H -5.62851500 2.21589100 0.60652900

H -4.83289300 0.99231500 0.06536500

H -4.41992600 -1.17163900 0.56313000

H -4.46579400 -1.10526600 -0.96839900

H -0.20489900 -2.68223800 0.09665100

H 0.83910700 -1.42677300 -0.15945700

H -0.52890200 3.10939900 1.75539700

H 0.62023600 2.20754200 2.28268800

H -3.19902600 2.56049500 0.69186700

H -1.94811500 2.95726700 -0.13725700

H -2.08439700 -2.88521500 -1.01078700

H -2.19253300 -2.91624400 0.52697300

H -2.43615500 -1.03475700 1.62106100

H -3.15317800 -1.97229200 2.65848700

H -2.55102300 -1.03917700 -2.24429100

H -3.30941800 -2.11719700 -3.09163500

H -1.48125300 1.14603000 -1.96023100

H -1.42459000 0.13864900 -0.78444300

H -0.49529700 0.15056300 1.51521000

H -1.66857500 1.09416600 1.05499800

H -0.10541000 2.91867100 -1.50687600

H -1.11499700 3.66501800 -2.45209300

H 3.80483400 -0.27789100 1.10926200

H 3.02140900 -1.58860800 1.41709400

H 4.30726700 3.63011500 -0.19342800

H 3.09336900 2.87250400 -0.78055600

H 4.67954400 1.31947300 -0.47146400

H 5.85778700 0.34405000 -0.16547700

H 2.00810400 -0.58281300 -1.60628200

H 0.44736900 -0.16937600 -1.75458100

H 3.19845000 -2.15077600 -1.36383800

H 4.06764400 -0.86624500 -1.45552100

H 1.80374400 0.03410900 2.20283600

H 0.87320000 0.04853400 3.44677200

H 1.35081300 1.52997100 -0.71346500

H 1.06017200 2.72410700 0.27394700

S 2.07549000 -3.32555700 0.36023300

$HOSH_{ice}$

O -1.04413400 2.98944100 -1.74973100

O 1.39554000 2.57691000 -0.68924100

O 1.24556700 -0.09025300 -1.22550000

O 3.58885800 -1.57194200 -1.90339600

O 0.47442300 3.06327100 1.81386100

O 0.96683100 0.40948800 2.55504600

O 3.22427600 -0.67073700 1.69916500

O 0.70089400 -2.29066900 0.39605800

O 4.12535300 2.91598200 -0.85033700

O 4.95284600 0.40796300 -0.21055600

O -1.48149300 0.22239800 -1.69935500

O -3.21938200 -1.73566700 -2.25977300

O -1.67426100 -3.19882300 -0.33300300

O -3.10570100 -1.82192200 1.67334000

O -1.38541500 0.15240500 1.09128100

O -2.21323200 2.72175800 0.81247200

O -4.76568000 1.98966600 0.31027800

O -4.90013300 -0.67573700 -0.18303900

H -5.61467800 2.25158300 0.66427900

H -4.83274400 1.03231400 0.09497200

H -4.44761800 -1.14922700 0.53246300

H -4.51633500 -1.03703600 -0.99592800

H -0.13481200 -2.69174900 0.03823900

H 1.14194400 -0.76777400 -0.53161400

H -0.48829500 3.12706500 1.74631000

H 0.65830400 2.21454200 2.25064800

H -3.18162900 2.58225300 0.74680200

H -1.92809200 2.96653100 -0.08350500

H -2.14217200 -2.86673200 -1.11442100

H -2.22954600 -2.93190200 0.41692400

H -2.47313100 -1.07064800 1.60494600

H -3.22050100 -2.02525000 2.60150700

H -2.61276800 -0.96406400 -2.25485400

H -3.39780100 -1.96084900 -3.17302700

H -1.51166600 1.17863800 -1.87471800

H -1.48494400 0.13704500 -0.72398200

H -0.51416400 0.11463800 1.53172400

H -1.67680900 1.09110600 1.12055800

H -0.09303700 2.95732100 -1.48832900

H -1.14541900 3.68093500 -2.40392100

H 3.84381000 -0.27243800 1.06130700

H 3.08101900 -1.58358200 1.40849300

H 4.37869500 3.66083500 -0.30451700

H 3.14605700 2.89646600 -0.84227200

H 4.71050100 1.32770400 -0.46108900

H 5.90010400 0.40087200 -0.07074600

H 2.79363400 -1.02781500 -1.98314500

H 0.37523700 -0.07300100 -1.65854300

H 2.58101900 -3.05165100 -0.66521600

H 4.20664400 -0.99407000 -1.43645600

H 1.81245400 0.04109500 2.18737000

H 0.92169400 0.09144100 3.45772000

H 1.40269200 1.59768300 -0.77297600

H 1.12020600 2.77703100 0.23628300

S 1.90568200 -3.42547400 0.44738300

H_2SO_{ice}

O -1.17852000 2.96469700 -1.81811400

O 1.22750200 2.51686200 -0.69446700

O 1.20167700 -0.12539100 -1.35426400

O 3.70770600 -1.32955000 -1.91616500

O 0.30585100 3.07570000 1.79660800

O 0.86166100 0.39617100 2.48864300

O 3.43221900 -0.71869900 1.90318600

O 0.88391800 -2.38626600 0.10206300

O 3.93195100 3.04645500 -0.63688300

O 4.95632400 0.58256600 -0.13281100

O -1.53824000 0.17597100 -1.83124400

O -3.20297800 -1.89885200 -2.12284900

O -1.71964200 -3.29697300 -0.08577100

O -2.98819100 -1.94049600 1.75410000

O -1.37889500 0.14945500 0.93825900

O -2.33933500 2.68564300 0.74737400

O -4.88892700 1.89677900 0.34605300

O -4.83918900 -0.78593900 -0.03005300

H -5.72587300 2.11162600 0.75601500

H -4.89785400 0.92826700 0.17461800

H -4.34994800 -1.22611100 0.68335500

H -4.47094000 -1.16192100 -0.84345600

H -0.77761700 -3.03815500 -0.08876700

H 1.08499300 -0.86676900 -0.71810800

H -0.65791800 3.10327400 1.70885900

H 0.50671400 2.25856400 2.27962900

H -3.30802600 2.53959000 0.70571700

H -2.07266700 2.93051100 -0.15449500

H -2.11665800 -2.96876700 -0.90663100

H -2.51496500 -2.56117200 1.13274800

H -1.99601400 -0.49371100 1.33492000

H -3.12796300 -2.40029500 2.58135200

H -2.63636100 -1.10314400 -2.22162400

H -3.42731900 -2.20513800 -3.00171700

H -1.61405900 1.12449200 -2.02608800

H -1.50995700 0.11937100 -0.85232100

H 0.12333600 0.15186200 1.88870400

H -1.78705700 1.04146800 0.99752700

H -0.23626900 2.91342100 -1.52891200

H -1.25109400 3.68194900 -2.44790300

H 3.96508800 -0.21879500 1.26435700

H 2.91336300 -2.37811700 1.15549500

H 4.05650600 3.73919600 0.01277600

H 2.96222500 2.93926000 -0.72727400

H 4.63739700 1.49311900 -0.32490400

H 5.91000100 0.63658700 -0.06461400

H 2.87055100 -0.85302000 -2.02219400

H 0.35140900 -0.09333900 -1.82129100

H 2.99428300 -2.76584400 -0.73219800

H 4.25102900 -0.72297700 -1.39094400

H 2.60330700 -0.22964900 2.02018000

H 0.67836600 -0.03469300 3.32499500

H 1.26596800 1.53974200 -0.79755500

H 0.93744900 2.70412200 0.22897300

S 2.18046500 -3.16874200 0.29946500

$TS1_{ice}$

O -1.03287300 2.89529200 -1.84161500

O 1.32774500 2.41696700 -0.61546400

O 1.20051200 -0.32371200 -1.11937700

O 3.23564300 -1.38530500 -1.78313800

O 0.39238900 3.08360900 1.80555600

O 0.93080000 0.46964000 2.58029000

O 3.23254300 -0.55193800 1.80173900

O 0.76132100 -2.14140900 0.40413900

O 4.02239200 2.84773000 -0.88879700

O 4.87765200 0.34216000 -0.44117600

O -1.41262900 0.11906700 -1.77312600

O -3.20044700 -1.85670300 -2.19011700

O -1.60760600 -3.24171700 -0.20253200

O -3.06278000 -1.78805200 1.74964500

O -1.34774400 0.15814300 1.01228700

O -2.24219800 2.70836500 0.72244200

O -4.78187500 1.93687300 0.25168300

O -4.87180600 -0.74122800 -0.13880600

H -5.62725600 2.19662300 0.61586900

H -4.83532100 0.97015800 0.07472400

H -4.41729200 -1.18214600 0.59631200

H -4.48123800 -1.12886400 -0.93633600

H -0.03568700 -2.66155300 0.13769000

H 0.94241800 -1.14350400 -0.37433000

H -0.56945700 3.13713300 1.71242600

H 0.57435500 2.26042200 2.29151500

H -3.20901800 2.54971400 0.66391000

H -1.96375300 2.93499300 -0.17942500

H -2.09454800 -2.95490200 -0.98841400

H -2.16209000 -2.96425200 0.54260300

H -2.42800700 -1.04825700 1.62069900

H -3.13286000 -1.95376300 2.68970400

H -2.58532800 -1.09640700 -2.24688400

H -3.40021400 -2.13743000 -3.08336900

H -1.46991300 1.06728900 -1.97991300

H -1.43208500 0.07129200 -0.79381100

H -0.49031800 0.13725600 1.48228700

H -1.66193100 1.08895200 1.03593300

H -0.10225900 2.84135400 -1.52481800

H -1.07997600 3.58779400 -2.50094600

H 3.83887800 -0.17563300 1.14942200

H 3.05194800 -1.46908100 1.48933500

H 4.29346800 3.57234700 -0.32426900

H 3.04566200 2.79669000 -0.81784900

H 4.65179900 1.28637400 -0.61800300

H 5.83279100 0.27882400 -0.40811600

H 2.22217000 -0.79246900 -1.59205400

H 0.42096800 -0.24838700 -1.69913400

H 3.06432800 -2.15793000 -1.13161300

H 3.94875200 -0.83590900 -1.38992500

H 1.80218000 0.12890100 2.23911200

H 0.83657600 0.08228400 3.45174800

H 1.31885000 1.44039500 -0.63841600

H 1.03043700 2.68253200 0.29139700

S 2.18341700 -3.17144400 0.40461800

$TS2_{ice}$

O 1.03790400 2.64801500 2.23223700

O -1.40787100 2.79590800 1.04084200

O -1.58750300 0.06677900 1.07016000

O -3.71232800 -1.64496100 1.38322300

O -0.22140600 3.45573600 -1.29300300

O -0.55017300 1.02516300 -2.67709400

O -2.36960400 -0.54244700 -1.55883800

O -0.63737200 -2.83223100 0.55738600

O -4.10796800 2.83392200 0.50317000

O -4.76442900 0.36248700 -0.39448600

O 1.13118800 -0.08386700 1.41326400

O 2.86999300 -2.01853900 1.89954100

O 1.61423000 -3.39507200 -0.09054900

O 2.58695300 -1.66284100 -1.78988900

O 1.30173100 0.32584600 -1.15089900

O 2.38232700 2.92066700 -0.28796200

O 4.88391400 1.82522500 -0.05381000

O 4.66629400 -0.88825800 -0.07324200

H 5.71210300 2.04216300 -0.48017400

H 4.82047000 0.84420900 -0.05198100

H 4.06221000 -1.18152300 -0.77867600

H 4.28393300 -1.26387300 0.73169600

H 0.38074400 -3.13590200 0.19444500

H -1.69182900 -0.16122800 0.12664100

H 0.73342300 3.41799800 -1.12016400

H -0.39473700 2.71515400 -1.89944800

H 3.32363600 2.66180600 -0.27492300

H 2.09152400 2.91163800 0.63557100

H 2.13462600 -3.10867000 0.69011600

H 1.95582600 -2.82143000 -0.83444100

H 2.04464800 -0.75894900 -1.58255100

H 2.69131600 -1.76297000 -2.73540000

H 2.22735500 -1.26339000 1.83847700

H 2.96844000 -2.24105100 2.82526800

H 1.24869700 0.79332600 1.81265700

H 1.19975300 0.06161400 0.41216200

H 0.23905600 0.69626300 -2.07124600

H 1.81728200 1.13902000 -1.05270400

H 0.11576300 2.83815200 1.94948500

H 1.19628900 3.13104400 3.04308100

H -3.24598400 -0.14836200 -1.42786800

H -2.03605300 -2.36666700 -1.20826600

H -4.28318000 3.59261300 -0.05449700

H -3.16906900 2.92018600 0.77681300

H -4.54971100 1.26098100 -0.04671000

H -5.66700600 0.40752800 -0.71154700

H -2.96128700 -1.05480000 1.59377200

H -0.66720700 -0.17987300 1.29811400

H -2.89173900 -2.74339600 0.49855200

H -4.27518900 -1.07552500 0.83140900

H -1.81188800 0.06246100 -2.10235800

H -0.26310900 0.96467800 -3.58831100

H -1.46058800 1.81585000 1.03457000

H -1.01216200 3.05537100 0.17145400

S -1.86208800 -3.34979500 -0.26771400
